# A peripheral primitive neuroectodermal tumor originating from the pancreas: a case report and review of the literature

**DOI:** 10.1186/s40792-015-0084-7

**Published:** 2015-09-11

**Authors:** Nobuyuki Nishizawa, Yusuke Kumamoto, Kazuharu Igarashi, Ryo Nishiyama, Hiroshi Tajima, Hiroshi Kawamata, Takashi Kaizu, Masahiko Watanabe

**Affiliations:** Department of Surgery, Kitasato University School of Medicine, 1-15-1 Kitasato, Minami-ku, Sagamihara, Kanagawa 252-0374 Japan

**Keywords:** Peripheral primitive neuroectodermal tumor, Ewing’s sarcoma, Pancreas, Small round cell tumor, Young adult, FISH

## Abstract

A peripheral primitive neuroectodermal tumor (pPNET) is a small round cell tumor occurring mostly in children or young adults and categorized into the Ewing sarcoma family of tumors. pPNETs originating from the pancreas are especially rare, and only 25 cases have been reported in the literature. We report a case of a 22-year-old man who had a giant expansive tumor located in the uncinate process of the pancreas, 80 mm in diameter resulting in obstruction in the duodenum. The patient underwent a pancreaticoduodenectomy. The histological examination showed that the pancreatic tumor was composed of atypical small round cells. Immunohistochemical findings were positive for CD99. An Ewing sarcoma breakpoint region 1 gene 22q12 rearrangement was proven by a two-color fluorescence in situ hybridization assay. We diagnosed the tumor as a pPNET of the pancreas, which, according to the literature, is highly aggressive with poor prognosis. A multidisciplinary approach to treat these neoplasms should improve the prognoses.

## Background

Peripheral primitive neuroectodermal tumors (pPNETs) are primary malignant neoplasms, usually occurring in children or young adults. These neoplasms are small round cell tumors arising from primitive neuroepithelial stem cells and categorized into the Ewing sarcoma family of tumors (ESFTs), which display common characteristics of morphology, histology, and genetics [1]. While the Ewing’s sarcoma is a primary bone tumor, pPNETs occur mostly in the soft tissue of the thoracopulmonary region, pelvis, and lower extremities [2]. pPNETs originating from the pancreas are extremely rare. To our knowledge, only 25 cases have been reported in the literature. Herein, we report a surgical case of a giant pPNET in the pancreas.

## Case presentation

A 22-year-old man, who presented with symptoms of upper abdominal discomfort and nausea during the previous month, was admitted to the emergency department of our hospital with extremely severe upper abdominal pain. He was obese with a BMI of 32.5. Laboratory data on admission showed slight anemia (hemoglobin 8.6 g/dL) but no elevation of bilirubin and low or normal amounts of several tumor markers, such as carcinoembryonic antigen (CEA), carbohydrate antigen 19-9 (CA19-9), and DUPAN-2. He did not show any metabolic abnormalities.

**Fig. 1:**
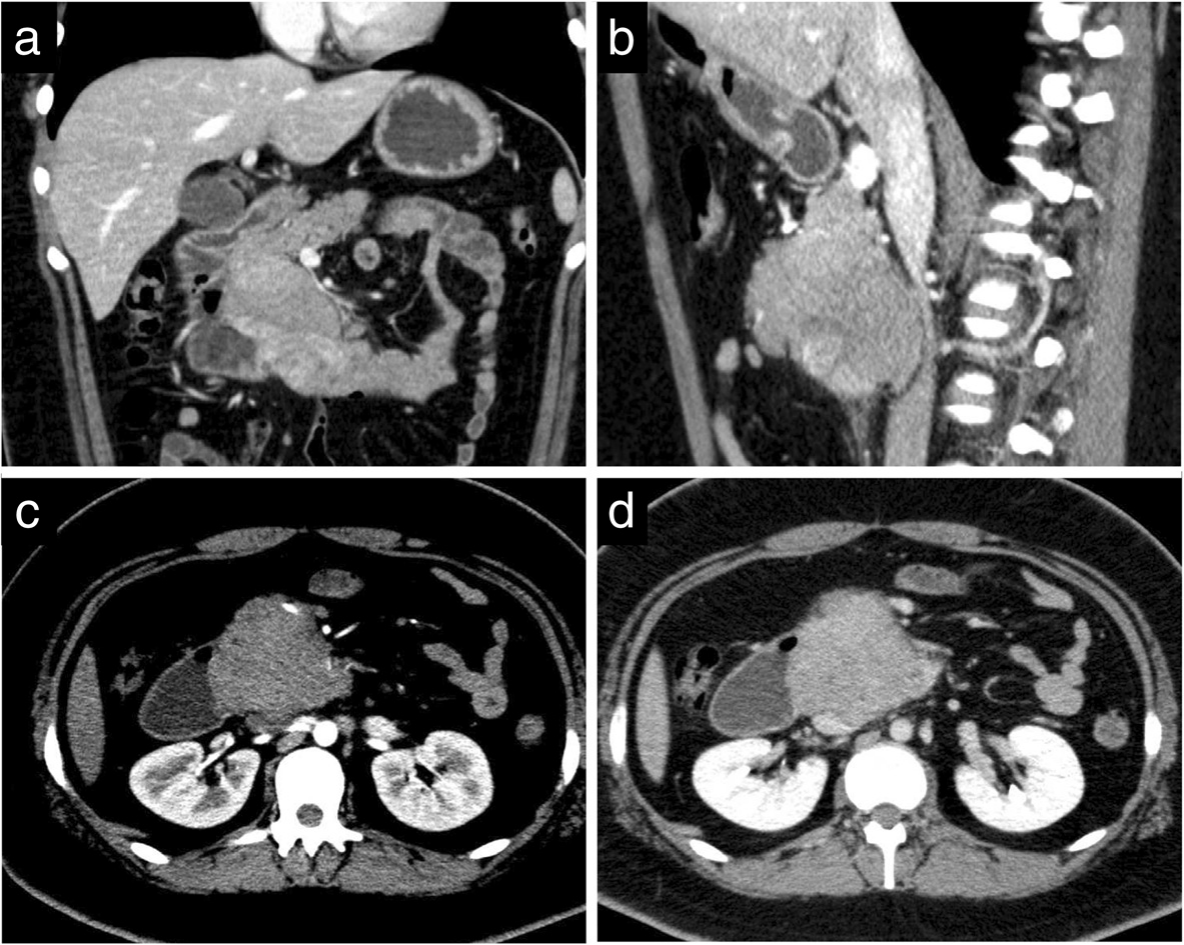
Contrast-enhanced CT of the abdomen. **a** The tumor was located mainly in the uncinate process of the pancreas and invaded the duodenum. The oral side of the duodenum was expanded. **b** The tumor excluded the inferior vena cava with no evidence of direct invasion. **c** The arterial phase: the tumor completely enclosed some intestinal branches. **d** The portal phase: there was no evidence of portal invasion

**Fig. 2:**
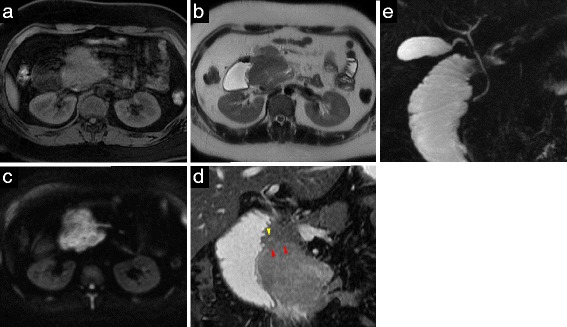
MRI of the abdomen. **a** T1-weighted images showed an isointense tumor compared with the pancreas. **b** T2-weighted images showed a slightly hyperintense homogenous tumor. **c** Diffusion-weighted images showed a hyperintense tumor. **d** FIESTA showed that the tumor edge (the *red arrow*) and the common bile duct in the pancreas (the *yellow arrow*) were located slightly distant. **e** MRCP showed that the common bile duct was not expanded and the main pancreatic duct was not depicted because it was too narrow

**Fig. 3:**
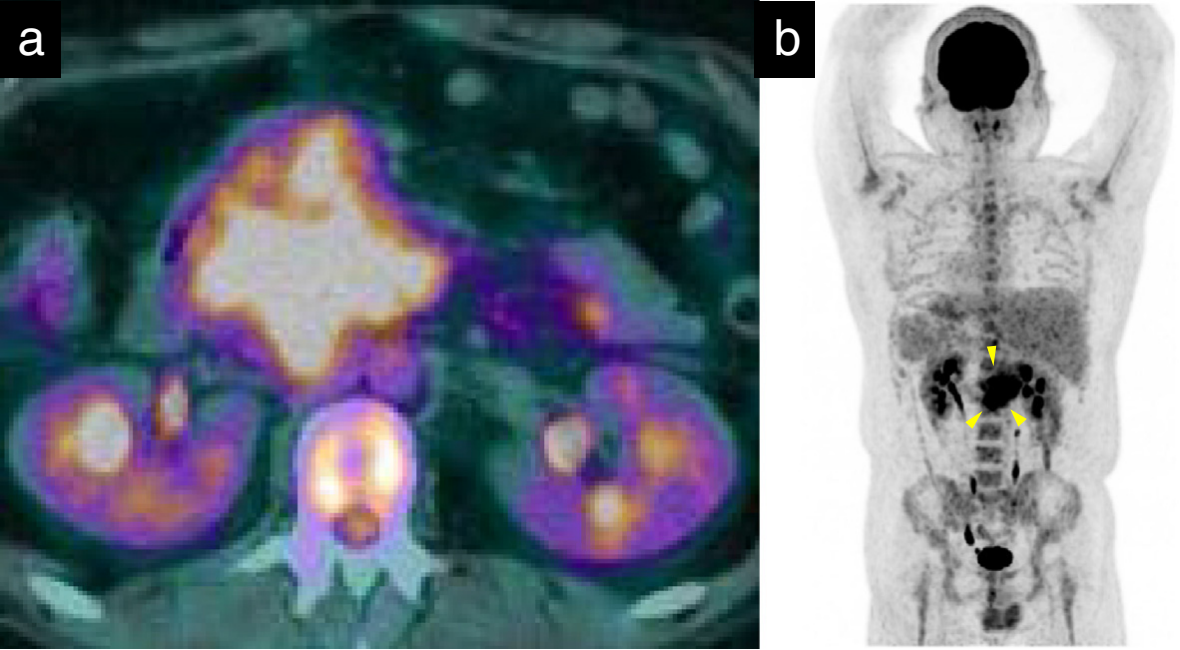
FDG-PET scanning of the abdomen and upper body. **a** The SUV max was 18.61. **b** There were no findings of metastases (*yellow arrows*)

Contrast-enhanced computed tomography (CT) scan revealed that a giant tumor 80 mm in diameter with mild enhancement occupied the pancreatic head. The tumor invaded the third portion of the duodenum, and the oral side of the duodenum was expanded. The superior mesenteric vein was shifted forward, and the inferior vena cava was squeezed by the expansive tumor (Fig. 1). Magnetic resonance imaging (MRI) showed that the tumor was homogenous and isointense on T1-weighted images (Fig. 2a) compared with the pancreas while slightly high intense on T2-weighted images (Fig. 2b). The tumor showed abnormal high intensity on diffusion-weighted images (Fig. 2c). Fast imaging employing steady-state acquisition (FIESTA) showed that the tumor was located slightly distal from the bile duct in the pancreas (Fig. 2d). In magnetic resonance cholangiopancreatography (MRCP), the common bile duct was not dilated and the main pancreatic duct was not depicted because it was too narrow (Fig. 2e). Positron-emission tomography with 18-fluorodeoxyglucose (FDG-PET) scanning showed a high accumulation of FDG in the tumor. The maximum standardized uptake value (SUV max) was 18.61, and there were no findings of metastasis (Fig. 3). Gastrointestinal endoscopy showed a gentle protuberance with mucosal reddening in the third portion of the duodenum, and the endoscope could not pass through. A biopsy was done from this region. At the same time, endoscopic ultrasound-guided fine needle aspiration was done. Based on cytohistological findings, acinar cell carcinoma or PNET was suspected; however, a definitive diagnosis was difficult because of inadequate samples. We planned an operation because it would be possible to accomplish complete resection of the tumor, which showed an expansive growth. We performed a pylorus-preserving pancreaticoduodenectomy (Whipple resection).

**Fig. 4:**
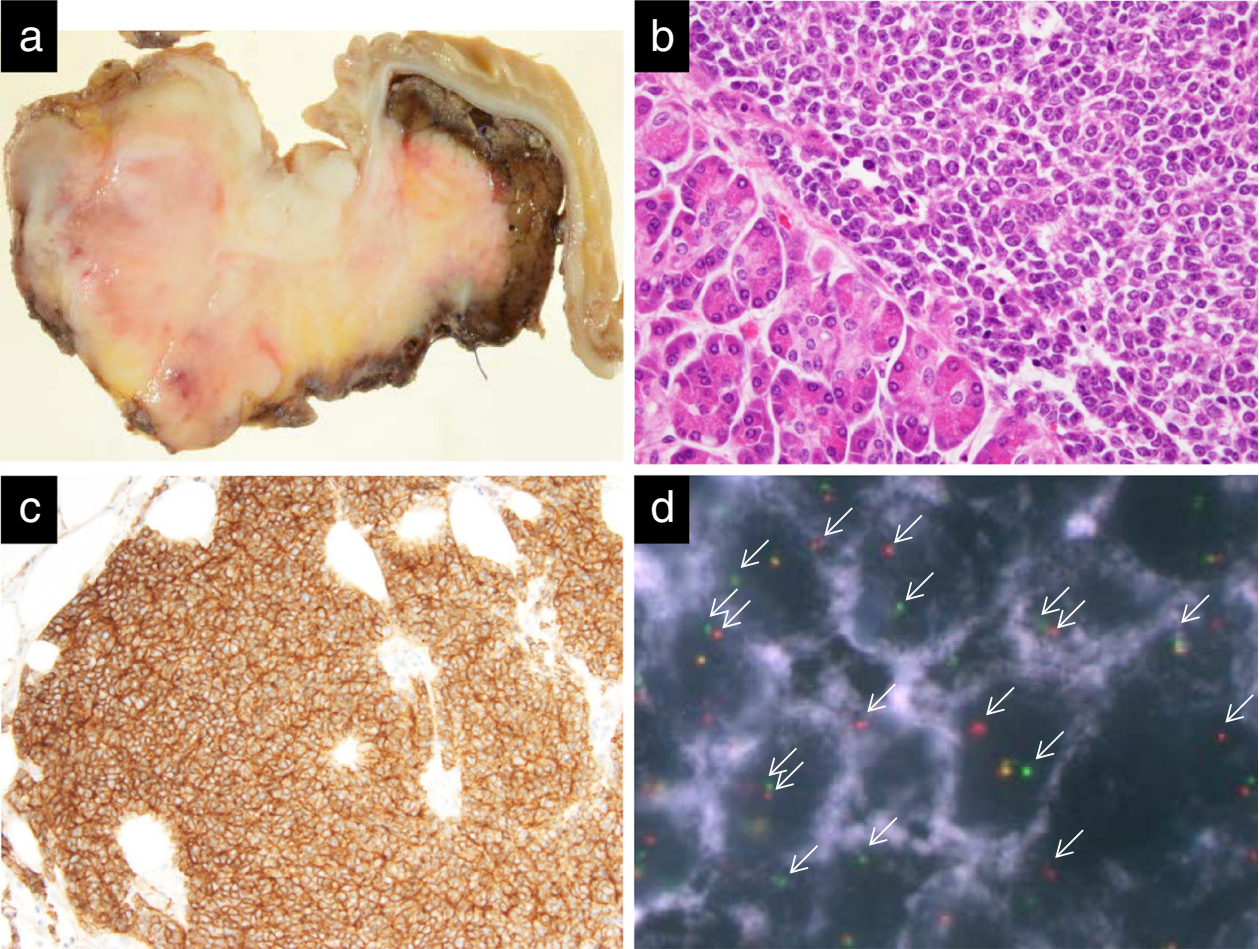
Pathologic findings. **a** Gross appearance of the cut surface of the tumor. **b** The tumor was composed of atypical small round cells with scant cytoplasm (hematoxylin and eosin staining, original magnification, ×100). The *left side* shows pancreatic acinar cells. **c** The tumor cells show strong cytoplasmic membrane positivity to CD99 (immunohistochemical staining, original magnification, ×100). **d** Two-color FISH assay results. The probe was localized to the breakpoints on chromosome 22q12 and provided evidence of the t(22q12) translocation by showing one *red* and one *green* signal pattern on the derivative chromosome 22

Macroscopically, the lesion presented as a light gray solid tumor which was 85 mm × 52 mm × 62 mm in size and occupied the uncinate process of the pancreas (Fig. 4a). Microscopic examination showed that the pancreatic tumor was composed of atypical small round cells with scant cytoplasm, and each had a round nucleus with a distinct nuclear membrane (Fig. 4b). The tumor cells invaded the duodenum and retroperitoneal fat tissue directly, and the retroperitoneal margin was histologically positive. There were aggressive lymphovascular invasions and lymph node metastases in 4 of 15 lymph nodes. Immunohistochemistry findings were strongly positive for CD99 (Fig. 4c), weekly positive for neuron-specific enolase, neural cell adhesion molecule, vimentin, synaptophysin, and CAM5.2, while negative for chromogranin A, cytokeratin AE1/AE3, cytokeratin 7, cytokeratin 20, carbohydrate antigen 19-9, CD10, and progesterone receptor. In addition, an Ewing sarcoma breakpoint region 1 gene, 22q12 rearrangement was proven by a two-color fluorescence in situ hybridization (FISH) assay (Fig. 4d). Finally, we diagnosed the tumor as a pPNET of the pancreas.

The patient developed postoperative complications such as a pancreatic fistula and a poor appetite and was treated conservatively. He recovered somewhat and was discharged 60 days after the surgery and transferred to a specialized facility for further adjuvant therapies.

### Discussion

pPNET was first described by Stout [3] in 1918 as a tumor of the ulnar nerve with the gross features of a sarcoma but composed of small round cells focally arranged as rosettes. On the other hand, Ewing’s sarcoma was first reported in 1921 by Ewing [4], which was an undifferentiated small round cell tumor that mostly occurred in the bones of children. However, recent advances of genetic investigation revealed that Ewing’s sarcoma and pPNET showed the same chromosomal translocations as t(11;22)(q24;q12), and both were classified into the same category of ESFTs by the World Health Organization Classification in 2002 [5]. ESFTs are made up of Ewing’s sarcoma, extraosseous Ewing’s sarcoma, Askin’s tumor, and pPNET. pPNETs account for approximately 1 % of all sarcomas [6] and 20 % of malignant soft tissue tumors in children [7]. pPNETs most often occur in soft tissues or bones. Although pPNETs seldom arise in organs, there have been some sporadic case reports of pPNETs arising in a variety of organs, such as the kidney, urinary bladder, lung, uterus, and vagina. Moreover, pPNETs arising in the pancreas are extremely rare, accounting for only 0.3 % of primary pancreatic neoplasms [8].

In reviewing the literature, 25 cases of pPNET of the pancreas including the present case have been reported to date (Table 1) [2, 8–24]. The mean age at diagnosis was 18.2 ± 9.6 (2–37 years) and the median was 20 years old, which was 12 years older than that in the retrospective study of 975 Ewing tumors of bones in Europe [25]. There was no sexual predominance in the 25 cases reviewed in the literature (13 males, 12 females). The most common presenting finding was abdominal pain (68 %), subsequently jaundice (20 %), nausea (16 %), and anemia (16 %). Endocrine disorders such as hyperglycemia and precocious puberty were accompanied in some cases. The tumor commonly occurred in the pancreatic head (68 %) and ranged in size from 35 to 220 mm (mean 88 mm), but supervened obstructive jaundice was seen in only 29 % because of its expansive growth, quite different from invasive growth like a ductal carcinoma.

**Table 1:** Clinical features of primitive neuroectodermal tumors of the pancreas

	Presenting finding										
Reference	Age	Sex	Upper abdominal pain	Other	Tumor location	Maximum tumor diameter	Diagnostic procedure	Lymph node metastasis	Treatment	Follow-up (month)	Outcome
Danner [9]	17	M	+	Jaundice, nausea	Head	90	Whipple resection	0/9	VDC/cisplatin + etoposide, RAD	33	NED
Luttges [8]	13	F	−	Diarrhea	Body	220	Whipple resection	NA	CHE	NA	NA
	31	M	+	−	Body	NA	Biopsy	NA	CHE	NA	NA
Bulchmann [10]	6	F	+	Anemia	Head	60	Whipple resection^a^	2/2	NP	6	DOD
O’Sullivan [11]	20	F	NA	−	Head	35	Whipple resection	4/34	CHE, RAD	30	AWD
Gemechu [12]	17	M	−	Abdominal swelling	Body	120	Resection	NA	NP	36	NED
Movahedi [2]	20	M	+	Jaundice	Head	35	Whipple resection	NA	NP	27	AWD
	25	F	+	Jaundice	Head	NA	Biopsy	NA	NA	NA	NA
	21	F	+	NA	Head	NA	Whipple resection	Positive	NP	DOC	DOC
	25	F	+	Jaundice	Head	80	Biopsy	NA	NA	NA	NA
	13	M	+	NA	Head	60	Biopsy	NA	VDC	43	NED
	6	M	+	Jaundice	Head	35	Whipple resection	Positive	VDC	48	DOD
Takeuchi [13]	10	F	+	Abdominal swelling	Body	100	Biopsy	NA	CHE, surgery, AST	3	DOD
Perek [14]	31	M	+	Fever, abdominal swelling	Head	120	Whipple resection	None	AI, ifosfamide, docetaxel	50	NED
Welsch [15]	33	M	−	Nausea and vomiting	Body	150	Distal pancreatecotomy^a^	NA	VIDE, VAI, melaphalan + etoposide, AST	12	NED
Schutte [16]	2	F	−	Precocious puberty	Body	60	Distal pancreatecotomy	None	VDC/AI	12	NED
Wakao [17]	3	M	+	Abdominal swelling	Head	82	Biopsy	NA	CITA, VDC/IE, MEC, surgery,^a^ AST, RAD (30 Gy)	8	NED
Doi [18]	37	M	−	Jaundice	Head	60	Whipple resection	Positive	VDC, IE, RAD	6	NED
Menon [19]	8	F	+	NA	Body	100	Biopsy	NA	Doxorubicin, RAD	19	DOD
Jing [20]	24	F	NA	NA	Head	100	Resection	NA	CHE, RAD	NA	NA
Maxwell [21]	11	M	−	Fatigue, anemia	Head	98	Whipple resection^a^	NA	VDC/IE	15	AWD
Mao [22]	13	F	+	Hyperglycemia	Head	150	Resection (RUPT)	None	VAC, MAID, RAD	41	AWD
Reilly [23]	23	M	+	Nausea	Body	58	Distal pancreatecotomy^a^	1/24	NA	NA	NA
Dias [24]	25	F	+	−	Head	42	Whipple resection	None	VAI, VDC	8	DOD
Present case	22	M	+	Nausea, anemia	Head	85	Whipple resection^a^	4/15	CHE, RAD	12	AWD

− absent, + present, *AI* actinomycin D (dactinomycin)/ifosfamide, *AST* autologous stem cell transplantation, *AWD* alive with disease, *CHE* chemotherapy (details unknown), *IE* ifosfamide/etoposide, *DOC* died of postoperative complication, *DOD* died of disease, *F* female, *M* male, *MAID* doxorubicin/dacarvazine/ifosfamide, *MEC* melphalan/etoposide/cisplatin, *NA* not available, *NED* no evidence of disease, *NP* not performed, *RAD* radiation, details unknown, *RUPT* resection of the uncinated process tumor, *VAC* vincristine/actinomycin D (dactinomycin)/cyclophosphamide, *VAI* vincristine/actinomycin D (dactinomycin)/ifosfamide, *VAIA* vincristine/doxorubicin/ifosfamide alternating with vincristine/actinomycin D (dactinomycin)/ifosfamide, *VDC* vincristine/doxorubicin/cyclophosphamide, *VIDE* vincristine/ifosfamide/doxorubicin/etoposide

^a^The tumor directly invaded another organ

Abdominal CT and MRI are the most useful modalities to reveal these tumors. Tan et al. [26] reported the radiographic characteristics of these tumors precisely, in which typical cases showed as isodense or hypodense on unenhanced CT, isointense on T1WI, and either isointense or hyperintense on T2WI as revealed by MRI. The tumors usually had ill-defined borders and irregular shapes with heterogeneous enhancement. The present case was consistent with these findings.

The usefulness of FDG-PET was not determined. Gyorke et al. [27] reported that FDG-PET was a valuable method for the diagnosis of ESFT and PNET. To the contrary, Doi et al. [18] reported that PNET showed a low level of SUV, and the detection sensitivity of FDG-PET was lower than that of helical CT. In the present case, the tumor showed a high level of SUV. An accumulation of more cases is needed to definitively determine the usefulness of FDG-PET for PNETs.

A histopathological examination is important for the diagnosis of pPNET. PNETs are small round cells and express the product of the *MIC2* gene on the X chromosome, which is confirmed with antibodies, such as CD99, O13, and 12E74. Moreover, another immunohistological analysis, such as neuron-specific enolase (NSE), vimentin, or cytokeratin, would be required to make a definitive diagnosis of pPNET. According to this review, a positive rate for NSE on pPNET was 92.9 % (13/14), that for vimentin was 88.9 % (8/9), that for cytokeratin AE1/AE3 was 57.1 % (8/14), and that for synaptophysin was 46.7 (7/15) (Table 2). The combination of these histological features and immune cell changes lead to an accurate diagnosis of pPNET.

**Table 2:** Immunohistochemical features of primitive neuroectodermal tumors of the pancreas

Reference	MIC-2 (CD99/O13/12E7)	NSE	AE1/AE3	VIM	SYN	CHR	Cytogenetic analysis	Chromosomal translocation
Danner [9]	+	+	+	NP	−	−	RT-PCR	EWS exon 7 to FLI1 exon6
Luttges [8]	+	+	+	+	−	−	NA	NA
	+	+	−	+	−	−	NA	NA
Bulchmann [10]	+	+	NP	−	−	−	FISH	EWSR1
O’Sullivan [11]	+	NP	−	+	NP	NP	RT-PCR	EWS exon 7 to FLI1 exon5
Gemechu [12]	NP	NP	NP	NP	+	+	NA	NA
Movahedi [2]	+	+	+	NP	−	−	RT-PCR	EWS-FLI1
	+	−	+	NP	−	−	NA	NA
	+	+	+	NP	+	+	RT-PCR	EWS-FLI1
	+	+	NP	NP	−	−	RT-PCR^a^	−
	+	+	−	NP	−	−	NA	NA
	+	+	+	NP	+	NP	RT-PCR	EWS-FLI1
Takeuchi [13]	+	+	+	NP	NP	NP	RT-PCR	EWS-FLI1
Perek [14]	+	NP	NP	+	+	−	^a^	−
Welsch [15]	+	+	+	+	+	NP	FISH	EWSR1
Schutte [16]	+	NP	−	+	+	+	NA	NA
Wakao [17]	+	NP	NP	NP	NP	NP	RT-PCR	EWS-FLI1
Doi [18]	+	+	−	+	NP	NP	FISH	EWSR1
Menon [19]	+	NA	NA	NA	NA	NP	NA	NA
Jing [20]	NA	NA	NA	NA	NA	NP	NA	NA
Maxwell [21]	+	NP	+	+	−	NP	RT-PCR	EWS-ERG
Mao [22]	+	+	−	NP	−	−	RT-PCR	EWS-FLI1
Reilly [23]	+	+	+	−	−	−	RT-PCR	EWS-FLI1
Dias [24]	+	NP	NP	NP	−	−	FISH	EWSR1
Present case	+	+	−	+	+	−	FISH	EWSR1

− absent, + present, *AE1/AE3* cytokeratin AE1/AE3, *CHR* chromogranin A, *EWSR1* Ewing sarcoma breakpoint region 1 gene one on 22q12, *FISH* fluorescence in situ hybridization, *NA* not available, *NP* not performed, *NSE* neuron-specific enolase, *RT-PCR* reverse transcript polymerase chain reaction, *SYN* synaptophysin, *VIM* vimentin

^a^RNA exhaustion from a paraffin block was impossible

The PNETs have a typical chromosome translocation involving *EWS* gene loci on chromosome 22q12, located in the 5′ sides of a chimera gene. Meanwhile, the 3′ sides of a fusion gene are known as *FLI1* t(11:22) (80–85 %), *ERG* t(21:22) (5–15 %), *ETV1* t(7:22) (rare), *E1AF* t(17:22) (rare), and *FEV* t(2:22) (rare) [28, 29]. *EWS* gene encodes a multifunctional protein that is involved in various cellular processes, including gene expression, cell signaling, and RNA processing and transport [30]. Chromosomal translocations encoding transcription factors result in the production of chimeric proteins that are involved in tumorigenesis. The detection of the chimera gene assures the diagnosis of pPNET and may be a useful prognostic factor [1]. There are two methods to detect a chimera gene: one is reverse transcript polymerase chain reaction (RT-PCR) and the other is FISH. In RT-PCR analysis, fresh or frozen tissues are better samples than formalin-fixed, paraffin wax-embedded (FFPE) tissues. Yamaguchi et al. [31] reported that the fusion transcripts could not always be detected by RT-PCR using FFPE tissue. Indeed, there were two reports that stated that the extraction of RNA could not be accomplished from formalin-fixed tissues. On the other hand, FISH analysis could be performed with FFPE tissues. The interphase FISH method using Ewing sarcoma breakpoint region 1 (EWSR1) dual-color, break-apart probes is sensitive and specific for the detection of rearrangement of the *EWS* gene on chromosome 22q12, although the probes specifically identify t(22q12) but cannot specifically identify the translocation partners [31]. The detailed examination of the chimera gene provides important information regarding prognosis [1]. Thus, in a case of an operation on a young patient suspected of a small round cell tumor or an undifferentiated tumor, it is recommended to preserve frozen samples of the tumor.

pPNETs are highly aggressive malignant tumors with almost inevitable recurrence and metastases. Metastases to the bone, bone marrow, lymph nodes, lung, liver, and other organs have been reported. Currently, the standard treatment of pPNETs is complete surgical resection with an adequate margin. Ozaki et al. [32] reported that the surgical resection for pPNET contributed to increase the disease control rate and survival rate, regardless of radiotherapy. However, with pPNETs originating from the pancreas, it is sometimes difficult to achieve complete resection with a safe surgical margin because unresectable organs, such as major vessels, are located adjacent to the pancreas. In the present case, the retroperitoneal margin was microscopically positive, as the anterior surface of the inferior vena cava could have been. Therefore, perioperative chemotherapy and radiotherapy play a great part toward controlling this type of disease.

## Conclusions

We reported a case of an extremely rare tumor originating from the pancreatic head in a young adult. When examining a young patient suspected of a small round cell tumor or undifferentiated tumor, frozen samples of the tumor should be used for a definitive diagnosis. Aggressive surgical resection in combination with chemotherapy and radiotherapy is the current standard of treatment, but the prognosis of this rare tumor remains unsatisfactory. To improve the outcome, the accumulation of such cases and further investigations are warranted.

## Consent

Written informed consent was obtained from the patient for publication of this Case report and any accompanying images. A copy of the written consent is available for review by the Editor-in-Chief of this journal.

## Abbreviations

CA19-9: carbohydrate antigen 19-9
CEA: carcinoembryonic antigen
CT: computed tomography
ESFTs: Ewing sarcoma family of tumors
EWSR1: Ewing sarcoma breakpoint region 1
FDG-PET: positron-emission tomography with 18-fluorodeoxyglucose
FFPE: formalin fixed, paraffin wax embedded
FIESTA: fast imaging employing steady-state acquisition
FISH: fluorescence in situ hybridization
MRCP: magnetic resonance cholangiopancreatography
MRI: magnetic resonance imaging
NSE: neuron-specific enolase
pPNET: peripheral primitive neuroectodermal tumor
RT-PCR: reverse transcript polymerase chain reaction
SUV max: maximum standardized uptake value
